# Supplementary material for: Dishevelled2 activates WGEF via its interaction with a unique internal peptide motif of the GEF
Source: Commun Biol. 2024 May 7;7:543. doi: 10.1038/s42003-024-06194-6 (PMC11076555; doi:10.1038/s42003-024-06194-6)
Supplement: Supplementary file 2 — Supplementary Information [file 42003_2024_6194_MOESM2_ESM.pdf]

Dishevelled2 Activates WGEF via its Interaction with a Unique Internal Peptide Motif of the GEF

Aishwarya Omble [1, 2], Shrutika Mahajan [1], Ashwini Bhoite [1, 2] & Kiran Kulkarni [1, 2, *]

[1]***Division of Biochemical Sciences, CSIR-National Chemical Laboratory, Dr. Homi Bhabha Road, Pune- 411008, India***

[2]***Academy of Scientific and Innovative Research (AcSIR), Ghaziabad- 201002, India***

*** Corresponding author:**

Email id: ka.kulkarni@ncl.res.in

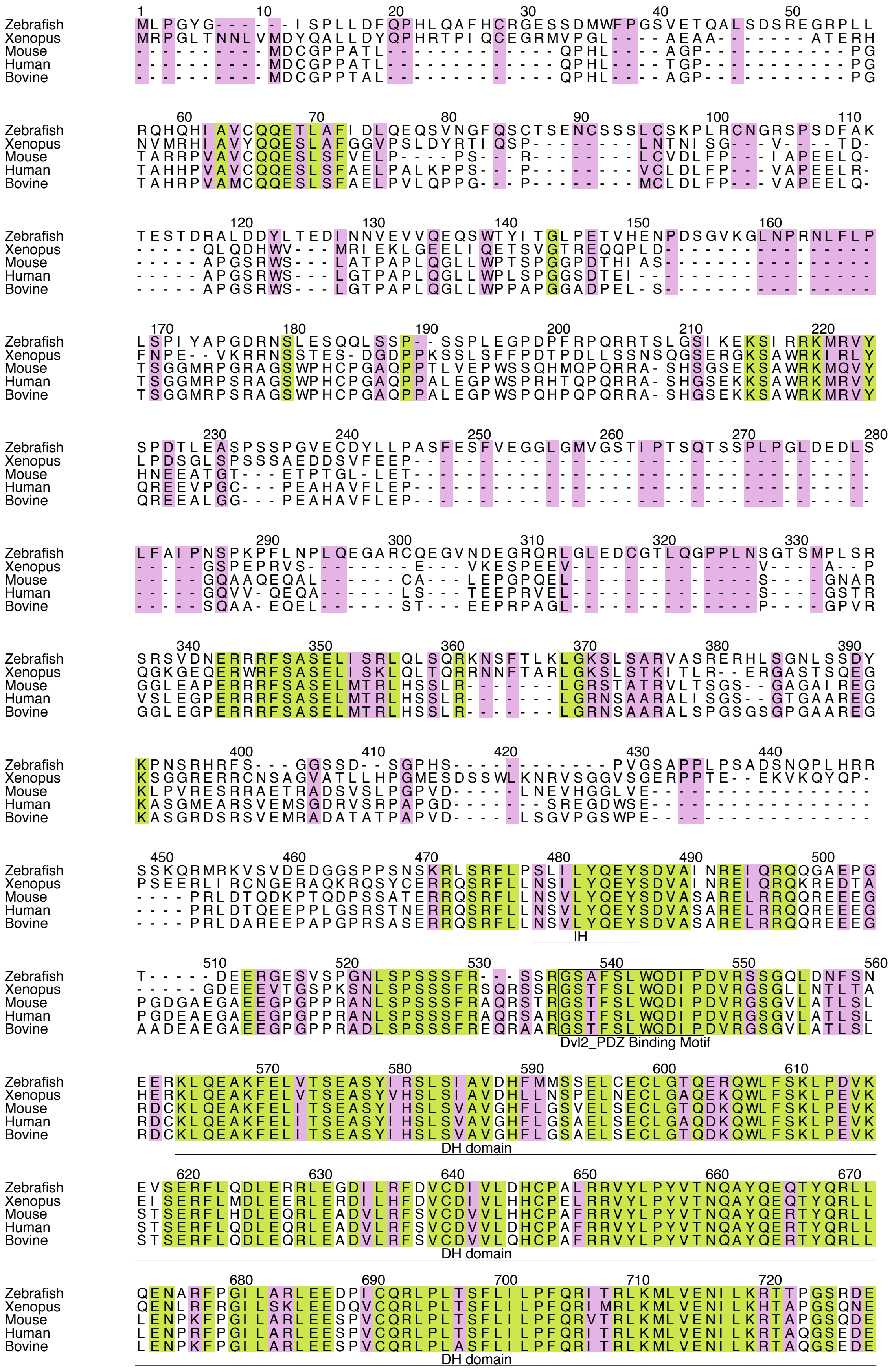
Zebrafish
Xenopus
Mouse
Human
Bovine
IH
Dvl2_PDZ Binding Motif
DH domain

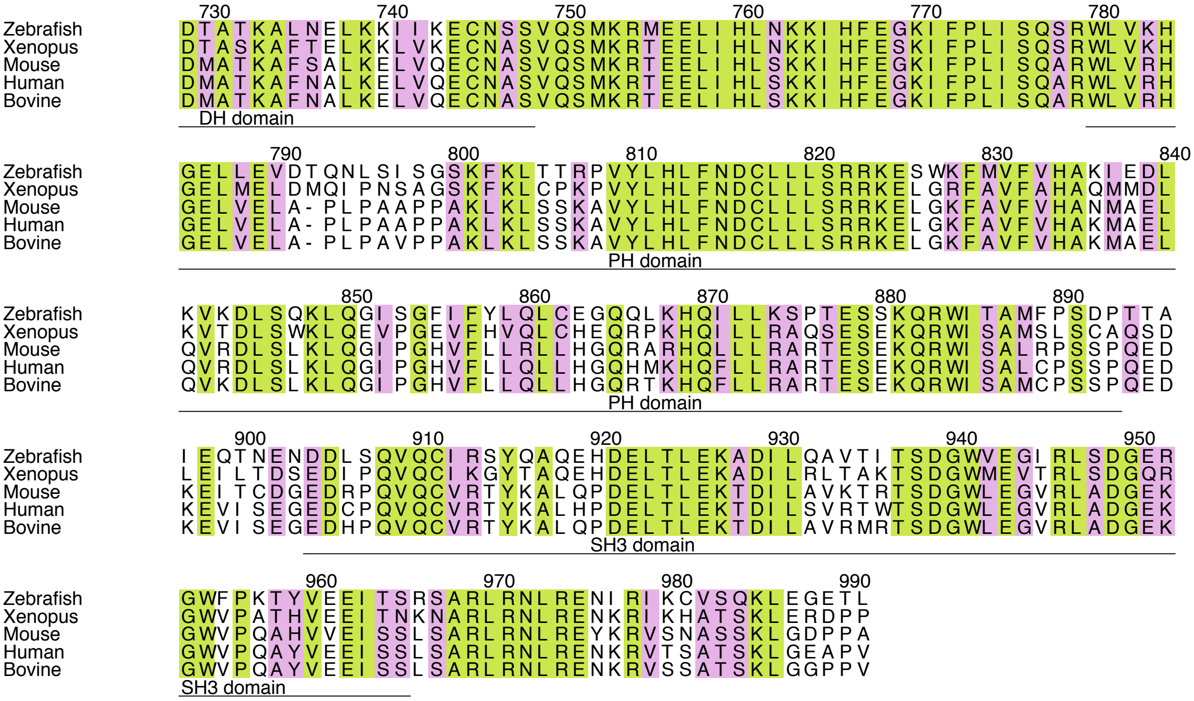

Supplementary Figure 1 Sequence alignment of WGEF from different organisms (Uniprot IDs: Zebrafish_E7EY47, Xenopus_A5X5J0, Mouse_Q8BWA8, Human_Q8IW93_1 and Bovine_E1BQ24). Sequences with 100% conservation along different organisms are highlighted in green, whereas sequences with 70% conserved sequence identity are highlighted in purple. The conserved $Dvl2^{PDZ}$ binding motif is marked with a black box, similarly, other regions like the N-terminal Inhibitory region (IH), the Dbl homology (DH) domain, Pleckstrin homology (PH) domain and a C terminal Src homology 3 (SH3) domain present in WGEF are labelled and underlined with a black line below the sequence.

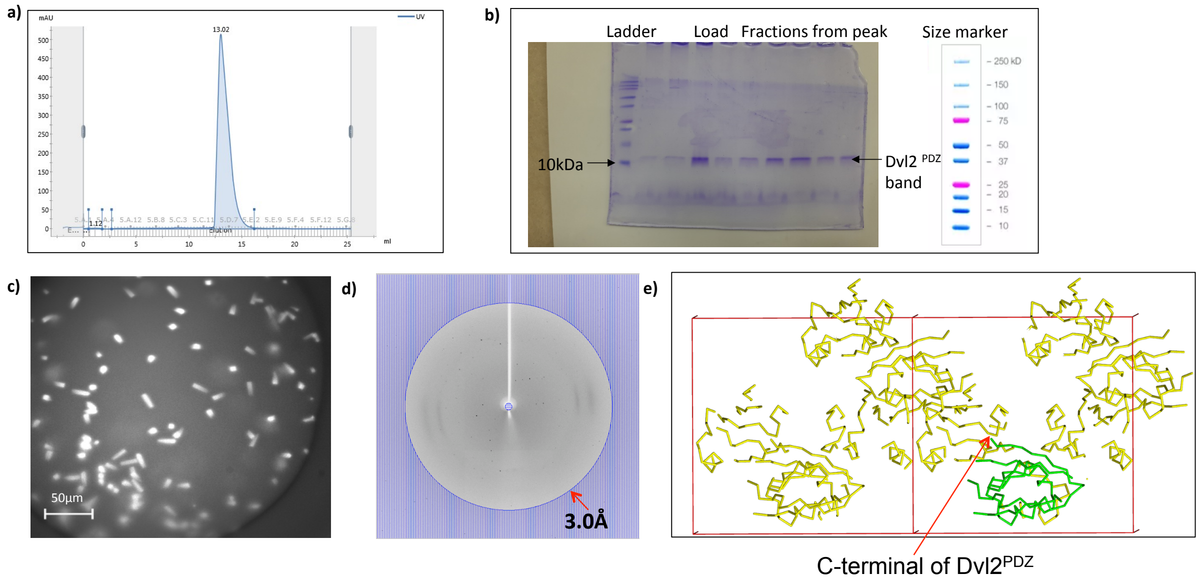

Supplementary Figure 2 Purification, crystallization, and structure determination of WGEF^{pep} fused Dvl2^{PDZ} **a)** gel filtration profile of WGEF^{pep} fused Dvl2^{PDZ} **b)** SDS PAGE image to show the purity of peptide fused Dvl2^{PDZ} **c)** Image showing WGEF^{pep} fused Dvl2^{PDZ} crystals under UV microscope **d)** Diffraction image showing diffraction upto 3Å **e)** The fused Dvl2^{PDZ} crystallized in I4_1 space group with ~0.6 solvent content. The crystal packing was found to be markedly different than the other reported peptide fused Dvl2^{PDZ} structures (PDB ID: 2REY, 3CC0, 3CBX, 3CBY, and 3CBZ). The electron density for the fused binding motif at the C-terminal of Dvl2^{PDZ} is not observed (PDB ID: 8YR7), as this particular segment of the structure is fully exposed to the bulk solvent. Furthermore, a very high concentration of the salt (3M NaCl) in the crystallization condition might have impeded the protein-protein interaction, resulting in the abrogation of interaction between the C-terminal peptide and the PDZ domain. Different constructs of Dvl2$^{PDZ\text{-}peptide}$ with varying linkers and configurations did not yield any crystals. The only construct that crystallized has a PDZ binding peptide (GSTFSLWQDIP) separated by four amino acid linker (SGGG).

a)

b)

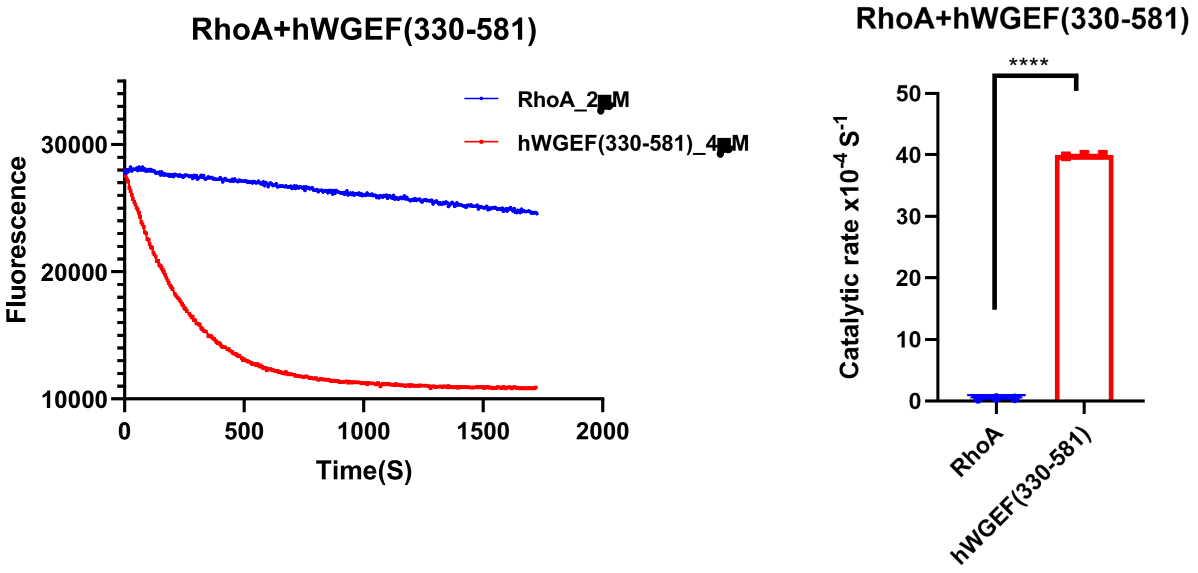

Supplementary Figure 3 Guanine nucleotide exchange assay of hWGEF (330-581). **a)** Nucleotide exchange curve of mant-GDP labelled RhoA (2μM) in the presence of hWGEF (330-581) (4μM). **b)** Bar graph showing catalytic rate of RhoA nucleotide exchange in the presence of hWGEF (330-581). Curves were plotted using GraphPad Prism. The error bar represents the standard deviation (± SD) obtained from triplicates. * Represents adjust *p* value with statistical significance of **** p <0.0001, *** p <0.001, ** p <0.01 and * p ≤0.01.

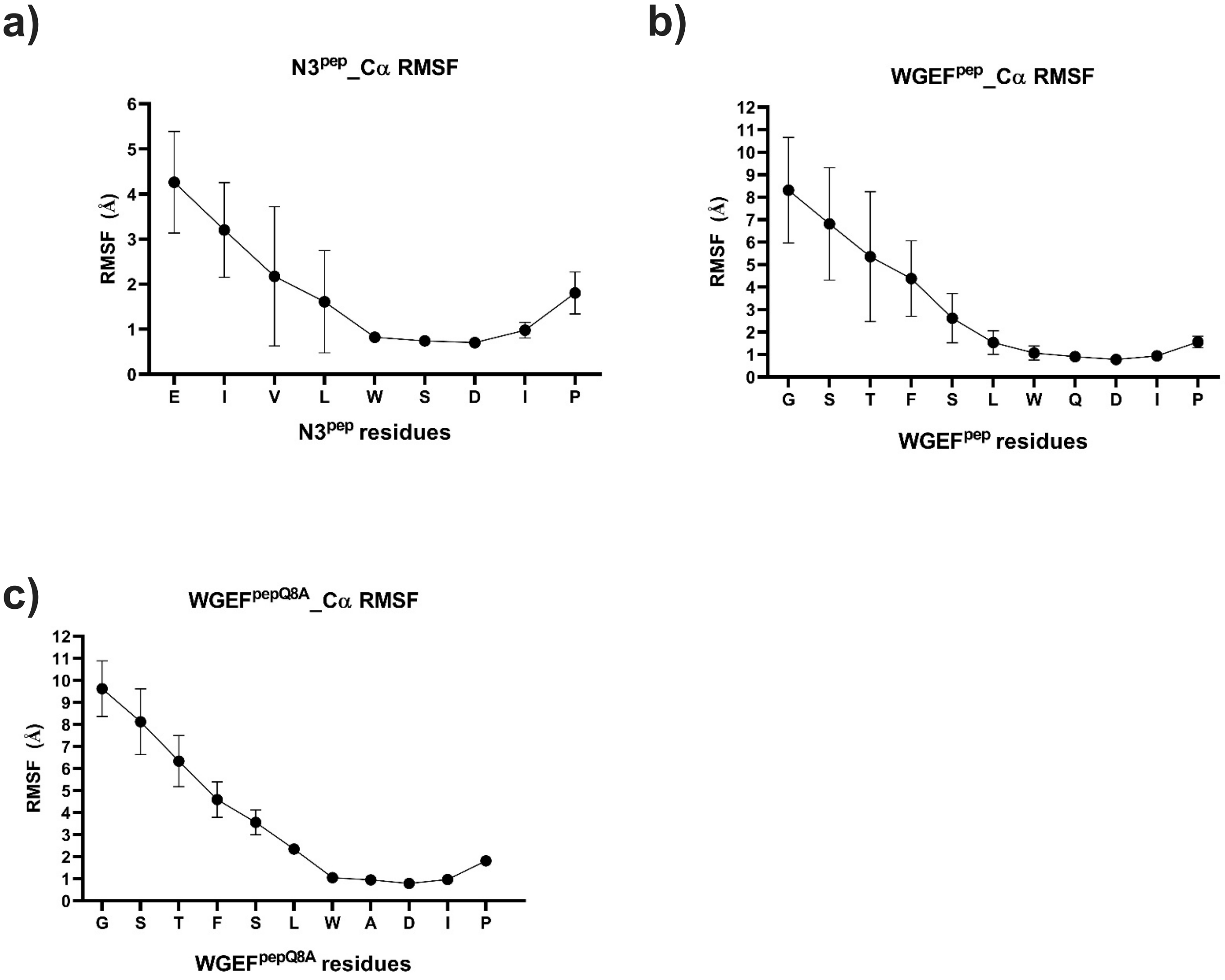

Supplementary Figure 4 Root-mean-square fluctuation (RMSF) plots of the Cα atoms of peptides from MD simulation studies with Dvl2^{PDZ}. **a)** RMSF of N3^{pep}, **b)** WGEF^{pep} and **c)** WGEF^{pepQ8A} used for studying different internal peptide–Dvl2^{PDZ} interaction employing MD simulations. Graphs were plotted using GraphPad Prism. Error bar denotes the standard deviation (± SD) calculated from three repeated runs.

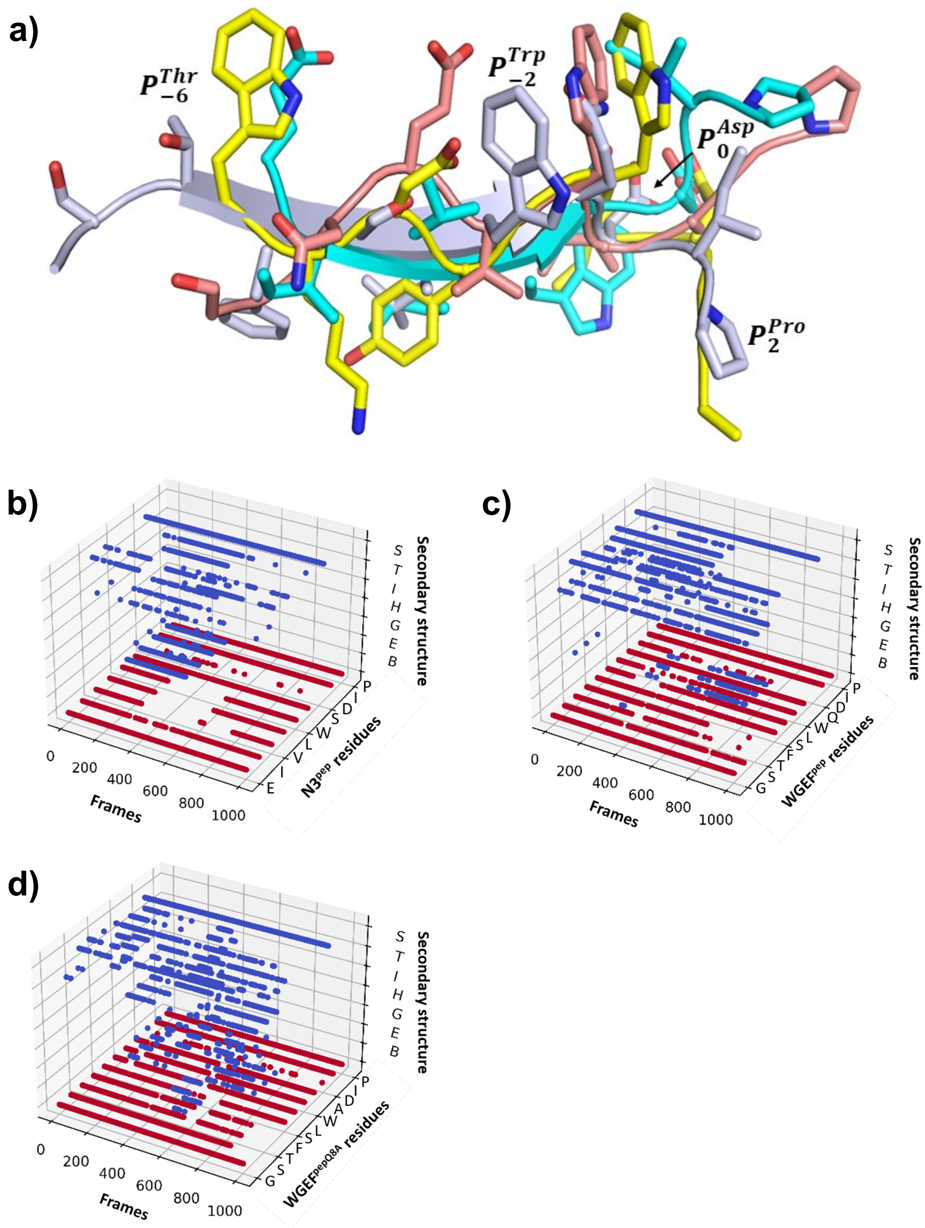

Supplementary Figure 5 Superimposition of internal peptides present in binding the pocket of Dvl2^{PDZ} and Secondary structural propensity of residues corresponding to the peptide. **a)** Yellow colour peptide represents N1 inhibitory peptide (PDB Code: 3CBY), pink colour peptide represents N2 inhibitory peptide (PDB Code: 3CBZ), blue colour peptide represents N3 inhibitory peptide (PDB Code: 3CC0) and grey colour peptide represents WGEF^{pep} peptide from one of the stable trajectories. Residue positions of WGEF^{pep} are labelled. **b)** Secondary structural probability of N3^{pep}, **c)** WGEF^{pep} and **d)** WGEF^{pepQ8A} residues during 1μs simulation of peptide–Dvl2^{PDZ} interaction. Numbers on the X-axis represent the No. of frames, Y-axis represents respective peptide residues and Z-axis represents secondary structure, where S= Bend, T= Turn, I= Helix-5, H= Alpha helix, G= Helix-3, E= Strand and B= Beta bridge. The presence of the specific secondary structure is indicated by a blue dot within its corresponding axis. Red color spots indicate the absence of secondary structure.

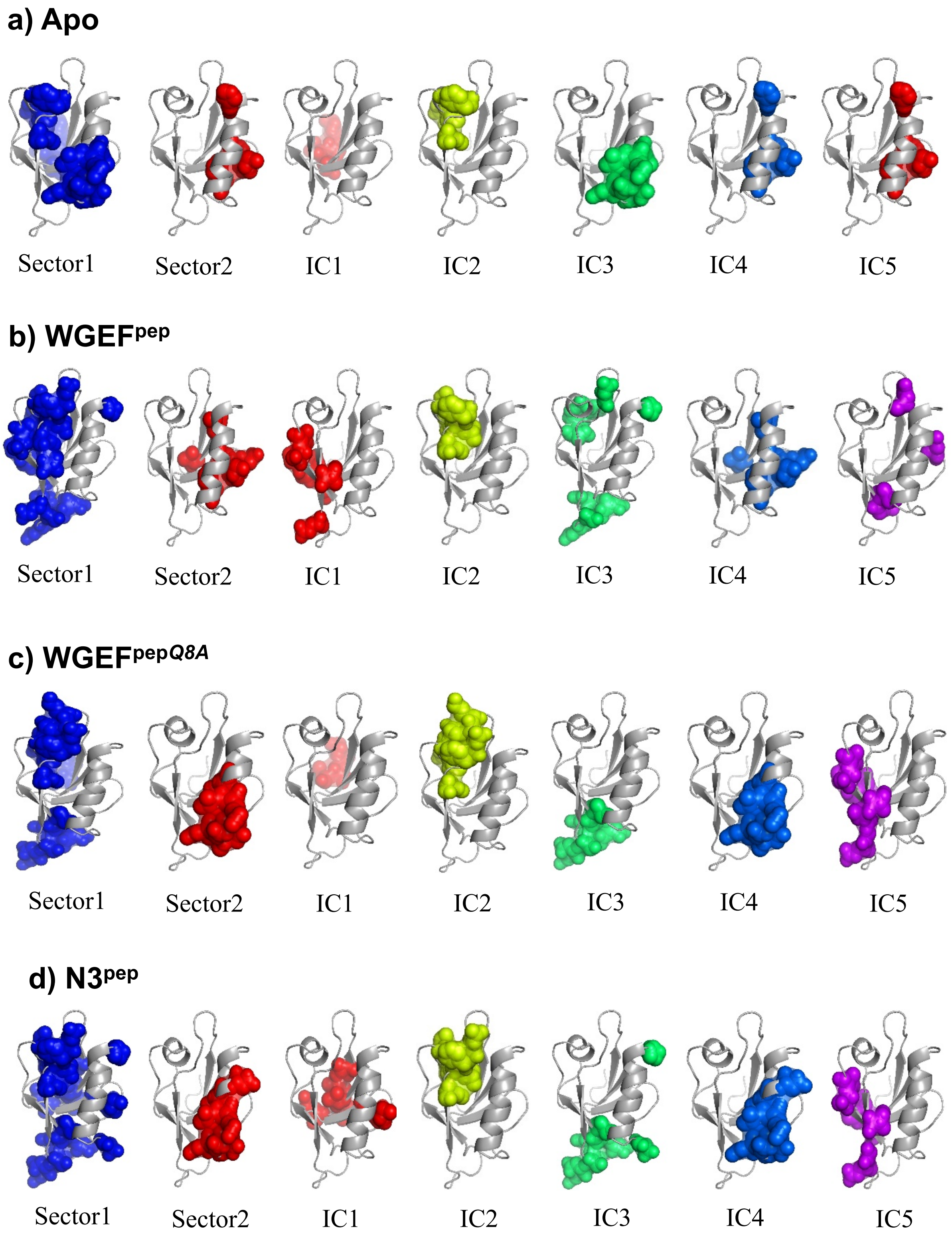

Supplementary Figure 6 Sectors and Independent components (ICs) mapped on Dvl2^{PDZ}. **a)** Sectors and ICs of *apo* Dvl2^{PDZ}, **b)** Dvl2^{PDZ} simulated with WGEF^{pep} **c)** Dvl2^{PDZ} simulated with WGEF^{pepQ8A} and **d)** Dvl2^{PDZ} simulated with N3^{pep}. Dvl2^{PDZ} structure is shown in cartoon, and residues corresponding to sectors and ICs are shown as spheres with different colours.

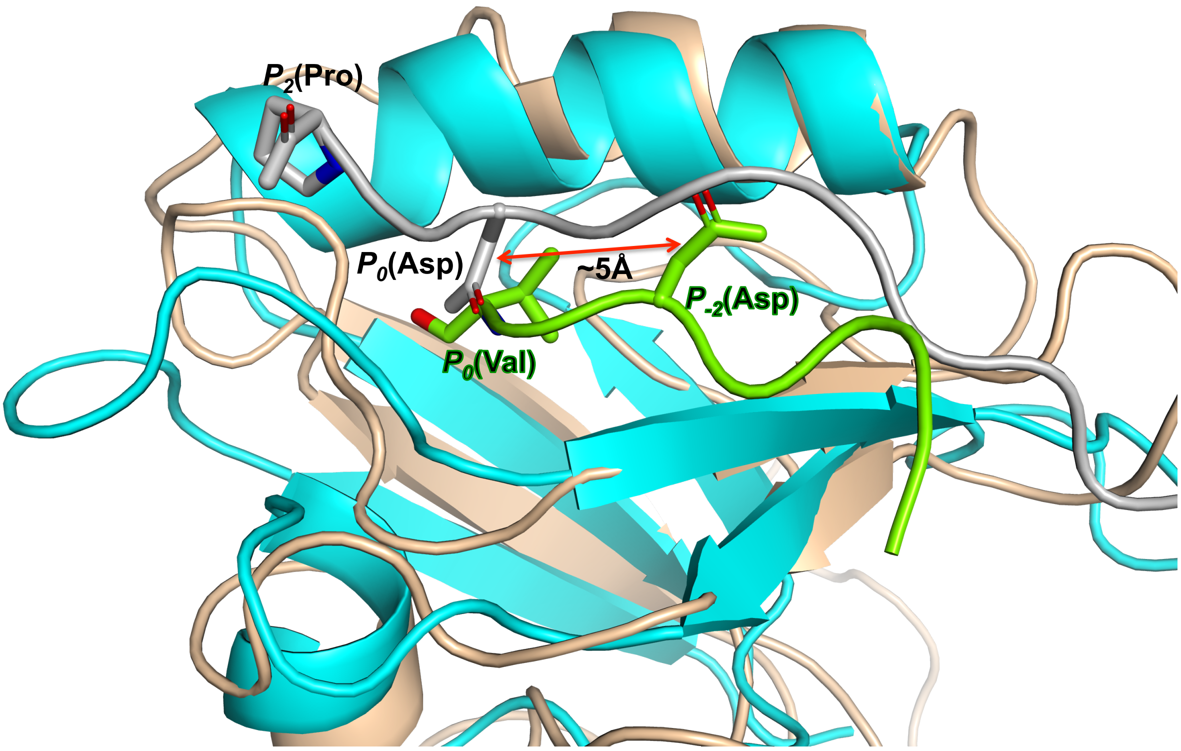

Supplementary Figure 7 Superimposition of Dvl2^{PDZ} (light brown) - internal peptide (grey) & nNOS^{PDZ} (cyan) - Class III peptide (green) (PDB:1B8Q) complex structures. The binding pocket of conserved Asp (P_0 in internal peptide and P_{-2} in Class III peptide) is shifted by ~5Å, thus indicating that the PDZ-peptide interaction in these two classes are highly divergent.

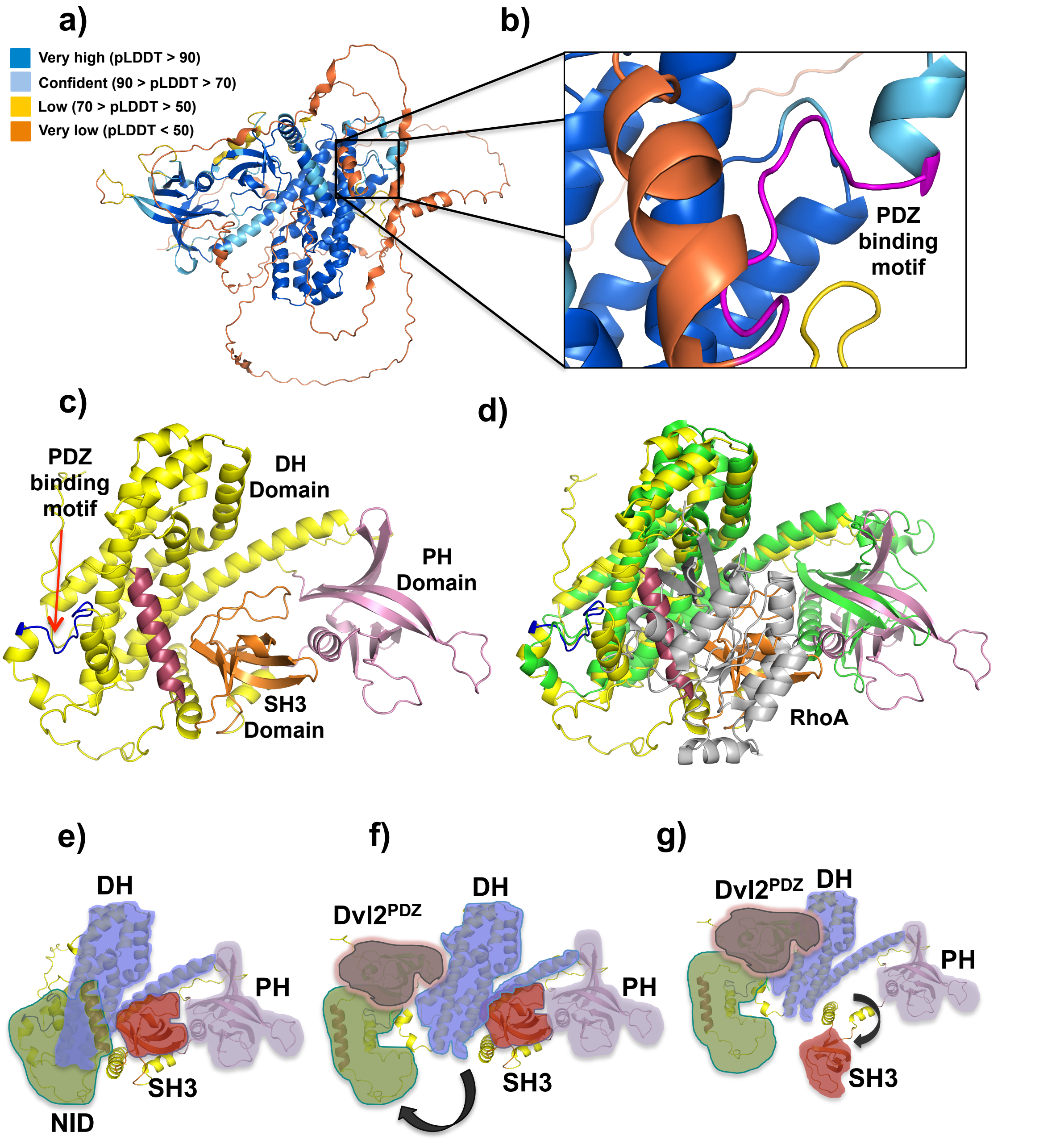

Supplementary Figure 8 hWGEF activation mechanism based on its AlphaFold structural model. **a)** Overall AlphaFold predicted structure of hWGEF. Regions of the structure are coloured based on per-residue confidence score (pLDDT). The level of increase in prediction confidence is scaled from red(low) to blue(high) **b)** The Dvl2^{PDZ} binding motif of hWGEF (shown in magenta) is zoomed **c)** Spatial disposition of DH(yellow), PH(pink), SH3(orange) and NID(brick) domains of hWGEF. The Dvl2^{PDZ} binding motif is shown in blue. **d)** Superimposition of hWGEF and Leukemia-associated RhoGEF (PDB ID: 1X86) structures. The latter is complexed with RhoA (shown in grey). **e)-g)** Putative mechanism of Dvl2^{PDZ} induced activation of hWGEF. Binding of Dvl2^{PDZ} to hWGEF could dismantle the intra-protein interaction, thereby unblocking the GTPase binding pocket of the GEF

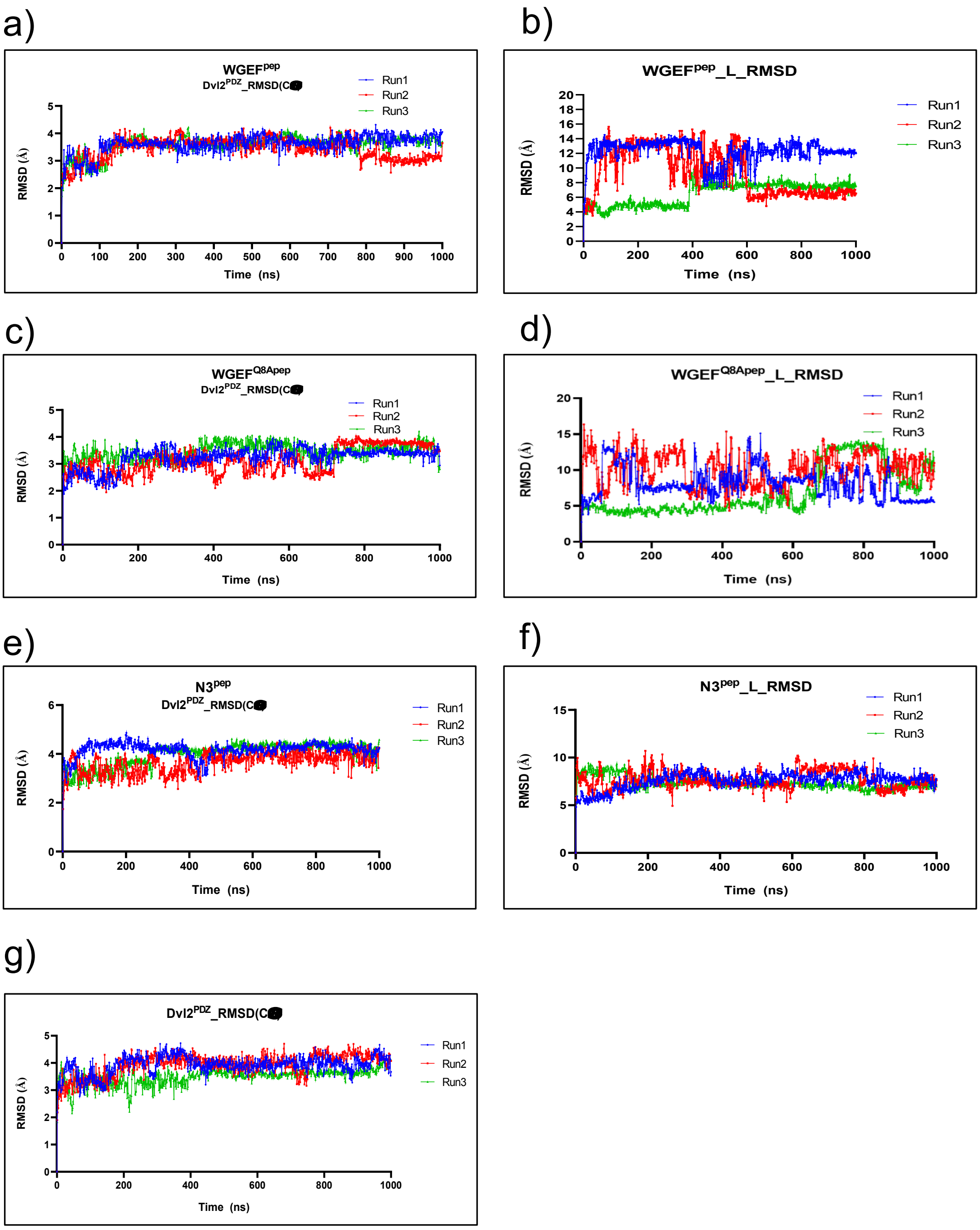

Supplementary Figure 9 Root Mean Square Deviation (RMSD) plots for comparing structural deviations of **a), c) and e)** Cα atoms of Dvl2^{PDZ} when simulated with WGEF^{pep}, WGEF$^{pep\mathit{Q8A}}$ and N3^{pep}, respectively **b), d) and f)** RMSD plots of peptides WGEF^{pep}, WGEF$^{pep\mathit{Q8A}}$ and N3^{pep} simulated with Dvl2^{PDZ} **g)** RMSD plot of *Apo* Dvl2^{PDZ}

Supplementary Table 1 Binding studies of Dvl2^{PDZ} with WGEF^{pep} variants, showing no detectable affinity between the WGEF mutant peptides (WGEF$^{pep\mathit{W7A}}$, WGEF$^{pep\mathit{D9A}}$ and WGEF$^{pep\mathit{P11A}}$) and Dvl2^{PDZ} protein.

Name:	**WGEF$^{pep\mathit{W7A}}$**	**WGEF$^{pep\mathit{D9A}}$**	**WGEF$^{pep\mathit{P11A}}$**
Graph Color:	●	●	●
Target Name:	PDZ	PDZ	PDZ
Target Concentration:	50 nM	50 nM	50 nM
Ligand Name:	WGEF$^{pep\mathit{W7A}}$	WGEF$^{pep\mathit{D9A}}$	WGEF$^{pep\mathit{P11A}}$
Ligand Concentration:	3.28 mM to 0.0001 mM	3.35 mM to 0.000102 mM	0.838 mM to 0.000102 mM
n:	3	3	3
Comments:			
Excitation Power:	20%	20%	20%
MST Power:	40%	40%	40%
Temperature:	24.2°C	22.7°C	24.2°C
Kd:			
Kd Confidence:			
Response Amplitude:			
TargetConc:	5E-08[Fixed]	5E-08[Fixed]	5E-08[Fixed]
Unbound:			
Bound:			
Std. Error of Regression:			
Reduced χ^2:			
Signal to Noise:			

Supplementary Table 2 MD simulation system parameters

**Parameters**	***Apo***	**N3^{pep}**	**WGEF^{pep}**	**WGEF^{pepQ8A}**
Number of simulations per system	3	3	3	3
Simulation box dimensions	10Å	10Å	10Å	10Å
Total number of atoms	17414	17772	18756	18761
Total number of water molecules	5323	5391	5713	5717
Simulation model	All atoms	All atoms	All atoms	All atoms
Force field	OPLS_2005	OPLS_2005	OPLS_2005	OPLS_2005
Water model	TIP3P	TIP3P	TIP3P	TIP3P
Solvent model	Explicit	Explicit	Explicit	Explicit
