# Supplementary material for: Dishevelled2 activates WGEF via its interaction with a unique internal peptide motif of the GEF
Source: Commun Biol. 2024 May 7;7:543. doi: 10.1038/s42003-024-06194-6 (PMC11076555; doi:10.1038/s42003-024-06194-6)
Supplement: Supplementary file 5 — Reporting Summary [file 42003_2024_6194_MOESM5_ESM.pdf]

nature portfolio

Corresponding author(s): Kiran Kulkarni

Last updated by author(s): Mar 20, 2024

Reporting Summary

Nature Portfolio wishes to improve the reproducibility of the work that we publish. This form provides structure for consistency and transparency in reporting. For further information on Nature Portfolio policies, see our Editorial Policies and the Editorial Policy Checklist.

Statistics

For all statistical analyses, confirm that the following items are present in the figure legend, table legend, main text, or Methods section.

n/a	Confirmed	
☐	☒	The exact sample size (*n*) for each experimental group/condition, given as a discrete number and unit of measurement
☐	☒	A statement on whether measurements were taken from distinct samples or whether the same sample was measured repeatedly
☒	☐	The statistical test(s) used AND whether they are one- or two-sided<br>*Only common tests should be described solely by name; describe more complex techniques in the Methods section.*
☒	☐	A description of all covariates tested
☒	☐	A description of any assumptions or corrections, such as tests of normality and adjustment for multiple comparisons
☒	☐	A full description of the statistical parameters including central tendency (e.g. means) or other basic estimates (e.g. regression coefficient) AND variation (e.g. standard deviation) or associated estimates of uncertainty (e.g. confidence intervals)
☒	☐	For null hypothesis testing, the test statistic (e.g. *F*, *t*, *r*) with confidence intervals, effect sizes, degrees of freedom and *P* value noted<br>*Give P values as exact values whenever suitable.*
☒	☐	For Bayesian analysis, information on the choice of priors and Markov chain Monte Carlo settings
☒	☐	For hierarchical and complex designs, identification of the appropriate level for tests and full reporting of outcomes
☒	☐	Estimates of effect sizes (e.g. Cohen's *d*, Pearson's *r*), indicating how they were calculated

Our web collection on statistics for biologists contains articles on many of the points above.

Software and code

Policy information about availability of computer code

Data collection	Diffraction data for the Dvl2PDZ-peptide complex and Dvl2PDZ-fused-peptide were collected at the ID23-2 beamline of the European Synchrotron Radiation Facility (ESRF), Grenoble, France and BL-21 PX beamline of the Indus-2 synchrotron at RRCAT, Indore, India, respectively.
Data analysis	All the data sets were integrated with XDS (doi: 10.1107/S0907444909047337) and scaled using AIMLESS (doi:10.1107/S090744491003982X), implemented on CCP4 software (https://doi.org/10.1107/S0907444910045749). Structure was solved by molecular replacement with PHASER(doi: 10.1107/S0021889807021206). The structure was further iteratively built using COOT (doi: 10.1107/S0907444910007493) and refined using PHENIX (doi: 10.1107/S0907444909052925).

For manuscripts utilizing custom algorithms or software that are central to the research but not yet described in published literature, software must be made available to editors and reviewers. We strongly encourage code deposition in a community repository (e.g. GitHub). See the Nature Portfolio guidelines for submitting code & software for further information.

Data

Policy information about availability of data

All manuscripts must include a data availability statement. This statement should provide the following information, where applicable:

- Accession codes, unique identifiers, or web links for publicly available datasets
- A description of any restrictions on data availability
- For clinical datasets or third party data, please ensure that the statement adheres to our policy

The atomic model of human Dvl2 PDZ crystallized in presence of WGEFpep and fused with WGEFpep are available in the Protein Data Bank (PDB) under the accession codes 8WWR & 8YR7, respectively. Initial coordinates, simulation input files and final coordinates of the MD simulation runs are available at https://zenodo.org/records/10683731. Source data used in the graphs,reported in this paper, are submitted to the journal as raw data (supplementary data 1). Additionally, reagents used in the study shall be shared upon request to the corresponding author.

Human research participants

Policy information about studies involving human research participants and Sex and Gender in Research.

Reporting on sex and gender	Not applicable
Population characteristics	Not applicable
Recruitment	Not applicable
Ethics oversight	Not applicable

Note that full information on the approval of the study protocol must also be provided in the manuscript.

Field-specific reporting

Please select the one below that is the best fit for your research. If you are not sure, read the appropriate sections before making your selection.

☒ Life sciences ☐ Behavioural & social sciences ☐ Ecological, evolutionary & environmental sciences

For a reference copy of the document with all sections, see nature.com/documents/nr-reporting-summary-flat.pdf

Life sciences study design

All studies must disclose on these points even when the disclosure is negative.

Sample size	All the biochemical assays were designed based on the structural models and MD simulations performed on these models.
Data exclusions	For biochemical assays data exclusion is not applicable. However, for the refinement of crystal structures against the diffraction data, 5% of the reflections were randomly excluded to measure the model bias and refinement validity in terms of Rwork and Rfree.
Replication	All the biochemical assays and MD runs were performed in triplicates.
Randomization	As explained above, 5% of the reflections from the diffraction dataset were randomly excluded to measure the model bias and refinement validity in terms of Rwork and Rfree.
Blinding	No clinical, ecological and behavioral data was involved in the study. Hence blinding of data is not applicable.

Reporting for specific materials, systems and methods

We require information from authors about some types of materials, experimental systems and methods used in many studies. Here, indicate whether each material, system or method listed is relevant to your study. If you are not sure if a list item applies to your research, read the appropriate section before selecting a response.

Materials & experimental systems

n/a	Involved in the study
☒	☐ Antibodies
☒	☐ Eukaryotic cell lines
☒	☐ Palaeontology and archaeology
☒	☐ Animals and other organisms
☒	☐ Clinical data
☒	☐ Dual use research of concern

Methods

n/a	Involved in the study
☒	☐ ChIP-seq
☒	☐ Flow cytometry
☒	☐ MRI-based neuroimaging
